# Effects of a Post-Deworming Health Hygiene Education Intervention on Absenteeism in School-Age Children of the Peruvian Amazon

**DOI:** 10.1371/journal.pntd.0003007

**Published:** 2014-08-14

**Authors:** François L. Thériault, Mathieu Maheu-Giroux, Brittany Blouin, Martin Casapía, Theresa W. Gyorkos

**Affiliations:** 1 Department of Epidemiology, Biostatistics and Occupational Health, McGill University, Montreal, Quebec, Canada; 2 Department of Global Health & Population, Harvard School of Public Health, Boston, Massachusetts, United States of America; 3 Division of Clinical Epidemiology, Research Institute of the McGill University Health Centre, Royal Victoria Hospital, Montreal, Quebec; 4 Asociacíon Civil Selva Amazónica, Iquitos, Perù; Instituto de Investigaciones en Enfrmedades Tropicales, Universidad Nacional de Salta, Argentina

## Abstract

Soil-transmitted helminth (STH) infections are a leading cause of disability and disease burden in school-age children of worm-endemic regions. Their effect on school absenteeism, however, remains unclear. The World Health Organization currently recommends delivering mass deworming and health hygiene education through school-based programs, in an effort to control STH-related morbidity. In this cluster-RCT, the impact of a health hygiene education intervention on absenteeism was measured. From April to June 2010, all Grade 5 students at 18 schools in a worm-endemic region of the Peruvian Amazon were dewormed. Immediately following deworming, nine schools were randomly assigned to the intervention arm of the trial using a matched-pair design. The Grade 5 students attending intervention schools (N = 517) received four months of health hygiene education aimed at increasing knowledge of STH prevention. Grade 5 students from the other nine schools (N = 571) served as controls. Absenteeism was measured daily through teachers' attendance logs. After four months of follow-up, overall absenteeism rates at intervention and control schools were not statistically significantly different. However, post-trial non-randomized analyses have shown that students with moderate-to-heavy *Ascaris* infections and light hookworm infections four months after deworming had, respectively, missed 2.4% (95% CI: 0.1%, 4.7%) and 4.6% (95% CI: 1.9%, 7.4%) more schooldays during the follow-up period than their uninfected counterparts. These results provide empirical evidence of a direct effect of STH infections on absenteeism in school-age children.

## Author Summary

Soil-transmitted helminths (STHs) are intestinal parasites that infect over 2 billion people in the world. Children infected with STHs often experience growth delays and perform poorly in school. However, the specific effects of STH infections on school absenteeism in children are not well understood. In this study, the authors followed Grade 5 students from 18 different schools, for four months. Students from all 18 schools were administered drugs at baseline to treat STH infections. Students from nine randomly selected schools then received weekly lectures and were given booklets on STH prevention, in an effort to prevent re-infection. Using a post-trial non-randomized analysis, the authors found that, while this health education intervention had no effect on overall absenteeism rates, Grade 5 students who had been re-infected during the four months following treatment had higher absenteeism rates than students who were uninfected after four months. These results contribute to a better understanding of the negative impact of STH infections on child health, showing that STH infections do increase the risk of absenteeism. These results also show that health education interventions, while powerful tools in the fight against STH infections, likely need to be implemented over longer periods of time to impact overall absenteeism rates. These findings are of interest to decision makers looking to implement and evaluate STH-targeted education programs.

## Introduction

Soil-transmitted helminth (STH) infections are the world's most important cluster of neglected tropical diseases and cause significant morbidity in school-age children (i.e. children aged between 5 and 14 years) [1]. It has recently been estimated that the intestinal parasites *Ascaris lumbricoides*, *Trichuris trichiura* and hookworms (*Ancyclostoma duodenale* and *Necator americanus*), respectively, infect 287 million, 194 million and 135 million school-age children around the world [2]. STH-infected children are at an increased risk of physical and intellectual growth impairments [3]. Globally, school-age children lose 16.7 million disability-adjusted life years (DALYs) to STH infections every year, a morbidity representing over 11% of the total disease burden afflicting this age group [4].

It has long been argued that STH infections could also increase school absenteeism [5], but little research has adequately investigated this association. A randomized controlled trial in Kenya found that a deworming intervention reduced absenteeism by at least 7 percentage points – a reduction of 25% of the baseline absenteeism rate – in school-age children [6]. This trial had a large sample size and strong methodology, and is often cited as evidence that STH infections have an important effect on absenteeism in school-age children [3], [7]. However, to date, such positive strong results have not been replicated. Only two other trials have measured the impact of deworming interventions on school absenteeism, and neither found significant treatment effects [8], [9]. These latter two trials had important methodological limitations, such as high attrition rates, selection bias and no sample size calculations, which might have led to underpowered analyses. An extensive review of all three trials has recently been published [10]. Unfortunately, the pooled effect measures reported in this meta-analysis are difficult to interpret because these trials measured absenteeism differently, and because these trials were conducted in student populations with different baseline STH infection prevalences. In addition, a systematic literature search found only seven observational studies investigating the effects of STH infections on absenteeism in school-age children [11]–[17]. Summarizing these results is difficult, as study quality was variable and results were discordant.

Deworming interventions have clearly been shown to be successful at reducing STH burden in school-age children [3]. A single dose of albendazole can reduce the *Ascaris*, *Trichuris* and hookworm burdens, measured in eggs per gram of feces, by up to 99%, 77% and 97% of their respective baseline values [18]. Similarly, a single dose of mebendazole can reduce these parasite burdens by up to 99%, 83% and 84% of their respective baseline values [18]. In regions where over 50% of school-age children are infected with at least one STH species, the World Health Organization (WHO) recommends treating all school-age children with a single dose of albendazole or mebendazole, once or twice a year, through school-based programs [19]. Health hygiene education is also recommended by WHO as an essential component of any school-based deworming intervention [20]. By promoting hygienic behaviours, health hygiene education programs could further reduce the risk of STH re-infection following deworming, and thus maximize the health impact of deworming interventions. Indeed, we have shown previously that a health education intervention was effective in increasing STH knowledge and in reducing the intensity of *Ascaris* infection [21]. The present study had two main objectives: 1) to assess the effect of a health hygiene education intervention aimed at reducing STH re-infection on absenteeism, and 2) to estimate the relationship between STH infection intensity and absenteeism in school-age children.

## Methods

### Ethics statement

This study was approved by the Research Ethics Board of the Research Institute of the McGill University Health Centre in Montréal, Canada and the Comité Institucional de Bioética of the Asociación Civil Impacta Salud y Educación in Lima, Peru. The trial is registered with clinicaltrials.gov (Registration number: NCT01085799). Written informed consent was obtained from each child's parent or guardian and written child assent was obtained from each child.

### Study location and study participants

This trial was conducted in Belén, a community of extreme poverty located on the banks of the Itaya River in the Peruvian Amazon. Belén is home to approximately 65,000 people, most of whom have limited access to potable drinking water. Lacking sewers and sanitation systems, and subjected to seasonal floods, Belén is highly STH-endemic. Recent surveys have found that up to 86% of Belén Grade 5 students are infected with at least one STH species [22]. Grade 5 children were selected as the study population because: 1) their mean age was expected to be 10 years, the mid-range of the highest STH risk group of school-age children (5–14 years of age), in terms of STH prevalence, intensity and burden of disease; 2) they are able to provide informed assent to participate in the study, to complete interviewer-administered questionnaires, and to provide adequate stool specimens on the day of the interview; 3) previous experience suggested a high response rate; and, 4) they would still be in primary school the following year for potential follow-up study.

### Trial design

A detailed description of the study design is available elsewhere [21]. Briefly, 18 elementary schools were paired, based on Grade 5 enrolment and geographical zone. In each of the nine pairs, one school was randomly assigned to the intervention arm of the trial, while the other served as a control. Between April and June 2010, Grade 5 students from intervention and control schools were dewormed with a single oral dose of 400 mg albendazole. In addition, a pre-tested interviewer-administered questionnaire was used to collect information on sociodemographic indicators and STH-related knowledge. Immediately following deworming, Grade 5 students attending intervention schools received four months of enhanced health hygiene education, while Grade 5 students attending control schools followed their usual curriculum, which did not include the health hygiene education intervention. The health hygiene education intervention consisted of booklets, posters, interactive activities and weekly lectures aimed at increasing students' knowledge of STH transmission and prevention. Grade 5 teachers from intervention schools also participated in workshops on STH transmission and prevention. Participating students provided single stool specimens at two timepoints: 1) before deworming and 2) four months post-deworming. Stool specimens were analyzed using the Kato-Katz method [23], and the intensity of STH infection was categorized according to species-specific WHO threshold values [20]. Moderate and heavy infections were combined into a single category to increase statistical power. The health education intervention was shown to be effective in increasing STH knowledge and in reducing *Ascaris* intensity of infection [21]. The effect size measures for the intervention on *Trichuris* and hookworm re-infection did not reach statistical significance.

### Absenteeism data

Absenteeism data for each school day from the date of deworming to the date of follow-up stool specimen collection was abstracted from Grade 5 teachers' attendance logs. On any given day, students with justified or unjustified absences were classified as absentees, while students who attended class for at least part of the school day were classified as attendees. Absenteeism was defined as the percentage of school days a given student was absent among the total number of days the school was open during the four-month follow-up period.

### Socioeconomic status

Each student was categorized in a socioeconomic status (SES) quartile, using an asset-based proxy constructed with principal components analysis [24] that explained 36% of the variance. This proxy included the following assets: house made of cement/bricks, electricity-wired house, a gas stove, a radio, and a television. The 1^st^ quartile was comprised of students with the highest SES scores while the 4^th^ quartile was comprised of students with the lowest SES scores.

### Objective 1: Effect of a health hygiene education intervention on absenteeism

The mean absenteeism rates of girls and boys were compared using *t-*tests. Similarly, the mean absenteeism rates of students in each of the four SES quartiles were compared using analysis of variance (ANOVA). Scatter plots and univariate linear regression were used to assess the relationship between age and absenteeism.

The mean absenteeism rates of students attending intervention schools were compared to those of students attending control schools using *t*-tests. To measure the effect of the health hygiene education intervention on school-level absenteeism while accounting for clustering by school, two hierarchical models were fitted. Both models are specified below:
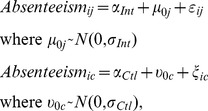
where *Absenteeism_ij_* is the absenteeism rate of student *i* in intervention school *j*; *µ_0j_* is a vector of school-specific random effects for the nine intervention schools; *ε_ij_* are the residuals in the intervention schools; *Absenteeism_ic_* is the absenteeism rate of student *i* in control school *c*; *υ_0c_* is a vector of school-specific random effects for the nine control schools; and *ξ_ic_* are the residuals in the control schools. Therefore, the intercepts of the first model (*α_Int_*) and second models (*α_Ctl_*) are the mean absenteeism rates in intervention and control schools, respectively. The effect of the education intervention on absenteeism rates was estimated by calculating the difference between the average absenteeism rates in intervention and control schools (i.e., *α_Int_* - *α_Ctl_*). Confidence intervals for this effect size measure were obtained from 9,999 bootstrap replicates. The clustered nature of our observations was taken into account by defining the resampling unit at the school level.

In the days immediately following deworming, the STH burden was expected to be very similar in both arms of the study. The health hygiene education intervention was hypothesized to delay re-infection in the intervention arm of the study. Therefore, the effect of the health hygiene education intervention on absenteeism rates was expected to become increasingly stronger over time (since deworming). To explore the sensitivity of our results, we repeated the analysis described above three times, excluding 1) the first 30 days immediately following deworming; 2) the first 60 days immediately following deworming; and 3) the first 90 days immediately following deworming.

### Objective 2: Effect of STH infection intensity on absenteeism

Three hierarchical linear regression models were built to estimate the effect of infection intensity on absenteeism, for each STH species separately (i.e. Model 1 for *Ascaris*, Model 2 for *Trichuris* and Model 3 for hookworm). The models used fixed effects for infection intensity, SES quartiles and age, and random effects at the school level. The three models had the following general formulation:
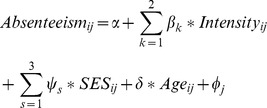
where *absenteeism_ij_* is the percentage of schooldays absent of student *i* from school *j*; *β_k_* is a vector of coefficients for light and moderate-to-heavy infections; *ψ_s_* is a vector of coefficients for the second highest, second lowest and lowest SES quartiles; *δ* is a coefficient for age; and *φ* is a school-level random effect. Similar models were also used to measure the effect of any STH infection and of STH infection by multiple species (i.e. polyparasitism) on the percentage of schooldays absent during the follow-up period.

The effect of *Ascaris* infections on absenteeism was also measured using a hierarchical logistic regression model. As previously stated, the single oral dose of 400 mg albendazole administered to the students is particularly efficacious against *Ascaris*, with up to 99% egg reduction rates [18]. It was therefore assumed that most students were nearly free of *Ascaris* infections in the days following deworming. Students who had heavier *Ascaris* infections at follow-up likely acquired these infections in the months following deworming. If *Ascaris* infections have a causal effect on absenteeism, the daily odds of absenteeism should increase with time in students who were infected after four months of follow-up, as these students gradually acquire their infections. Absenteeism was therefore redefined as a binary variable for each day, from the date of deworming to the date of follow-up stool specimen collection. The *logit* of absenteeism was modelled as a function of the number of days since deworming, *Ascaris* infection intensity at follow-up and SES quartile. Product terms between time since deworming and both *Ascaris* infection intensity at follow-up and SES quartile were added to the model. Random intercepts were also added to the model to account for school clustering and for within-student correlation in daily observations. Scatter plots were used to visually assess the assumption of a linear relationship between the logit of absenteeism and the number of days since deworming. The model is defined as:
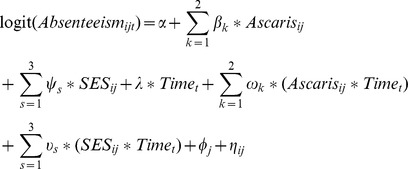
where *Absenteism_ijt_* is the probability of being absent for student *i* from school *j* on day *t*;

*β_k_* is a vector of coefficients for light and moderate-to-heavy infections; *ψ_s_* is a vector of coefficients for the second highest, second lowest and lowest SES quartiles; *λ* is a coefficient for time since deworming; *ω_k_* is a vector of coefficients for the interactive effect of *Ascaris* infection intensity and time; *υ_s_* is a vector of coefficients for the interactive effect of SES quartile and time; *φ* is a school-level random effect; and *η* is a student-level random effect.

Unless otherwise stated, all statistical analyses were performed using the R statistical software, version 2.11.1 [25]. The hierarchical models were fitted using the *lme4* library [26]. When applicable, histograms and residual plots were used to visually assess the distributional assumptions of the models used.

## Results

### Description of study population

**Figure 1:**
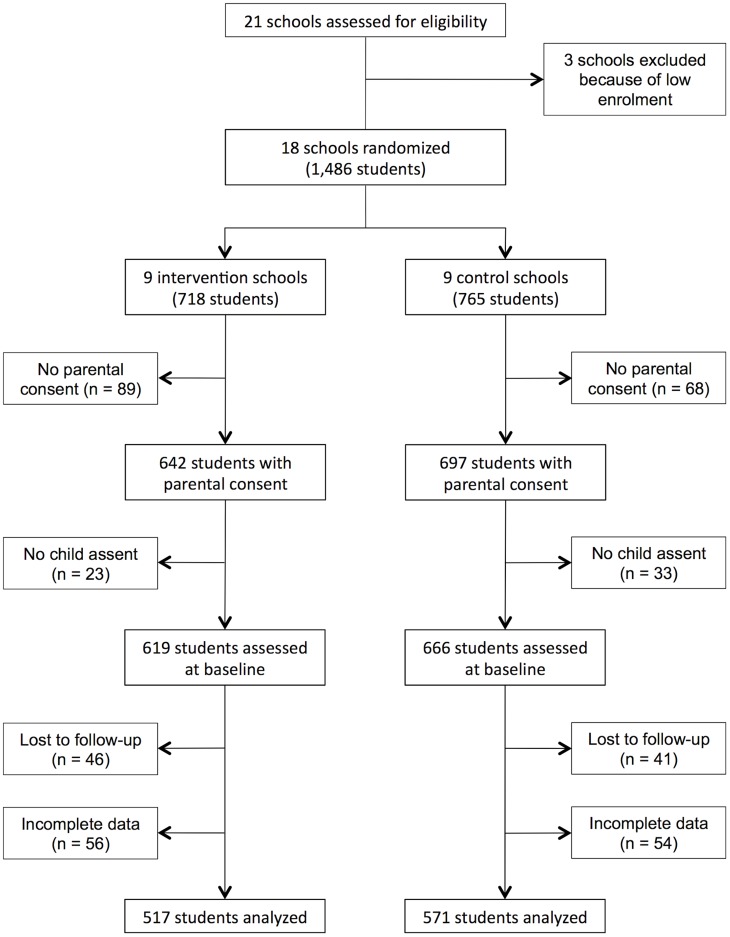
Trial flow diagram.

A total of 1,486 students were officially enrolled in Grade 5 at the 18 participating schools. Parental informed consent and child assent was provided for 1,285 students. Complete parasitological and absenteeism data were available for 1,088 students

(N = 517 in intervention schools and N = 571 in control schools) (Figure 1). The baseline characteristics of Grade 5 students did not differ significantly between intervention and control schools (Table 1). A detailed comparison of students in both arms of the trial is available elsewhere [21].

**Table 1:** Summary data showing the comparability in baseline characteristics of intervention and control study schools and children (adapted from [21]).

Level	Characteristics	Intervention	Control
Child-level characteristics	Number of children	517	571
	Age (mean (sd))	10.2 (1.1)	10.4 (1.1)
	Girls (%)	46.0	50.8
	Have been dewormed in the past (%)	64.7	63.6
	Number of people in the household (mean (sd))	7.0 (2.9)	7.3 (3.2)
	Any STH infection (%)	72.1	78.1
School-level	Number of schools	9	9
	Located in a seasonally flooded area (%)	44.4	44.4
	Total number of children in the school (mean (sd))	516.7 (384.8)	551.3 (421.8)

The 18 participating schools were open for a total of 85,107 student-days between the date of deworming and the date of follow-up stool specimen collection for the 1,088 Grade 5 students included in the final sample. Absenteeism data were available for 73,098 (85.9%) of these student-days. On average, students were absent for 11.3% (95% CI: 10.7%, 12.0%) of school days during the four-month follow-up period.

### Sex

Sex was not associated with absenteeism. On average, girls and boys were respectively absent for 11.3% and 11.3% of schooldays during the follow-up period (*p* = 0.97).

### Socioeconomic status

SES was significantly associated with absenteeism. On average, students in the highest, second, third and lowest SES quartiles were, respectively, absent for 9.4%, 11.1%, 12.8% and 13.5% of schooldays during the follow-up period (*p*<0.001).

### Age

Absenteeism tended to increase with age. Students' ages ranged from 8 to 16 years, with an average of 10.34 years. A hierarchical linear regression model that accounted for clustering at the school level estimated that absenteeism increased by 1.7% (95% CI: 1.1%, 2.3%) for every one-year increase in age.

### Objective 1: Effect of a health hygiene education intervention on absenteeism

The health hygiene education intervention had no statistically significant effect on absenteeism in Grade 5 children. On average, students attending intervention and control schools were respectively absent for 11.4% and 11.3% of schooldays during the follow-up period (*p* = 0.93). The mean absenteeism rate of intervention and control schools was estimated at 12.9% (95% CI: 10.2%, 15.6%) and 12.1% (95% CI: 9.1%, 15.0%), respectively. Attending an intervention school was not associated with a significant school-level decrease in absenteeism during the four-month follow-up period (difference  = 0.8%; 95% CI: −3.1%, 4.8) %).

Results were not statistically significantly different when the first, first two or first three months of data immediately following deworming were excluded from analysis. However, these results are consistent with the hypothesis that the magnitude of the health hygiene education intervention on absenteeism rates increases with time since deworming (Table 2). After removing the first three months of data immediately following deworming, attending an intervention school was associated with a slight, non-significant decrease in absenteeism (difference  = −0.8%, 95% CI: −4.5%, 2.9%).

**Table 2:** Sensitivity analysis: Mean percentage of schooldays lost to absenteeism in intervention (n = 9) and in control (n = 9) schools in the first 125 days following mass deworming, as estimated by the hierarchical models, which each exclude a different number of days post-deworming.

	Number of days immediately following deworming that are excluded from the analysis			
School	None	First 30 days	First 60 days	First 90 days
Intervention	12.90% (10.24%, 15.55%)	13.78% (11.13%, 16.43%)	13.09% (10.42%, 15.76%)	13.32% (11.01%, 15.63%)
Control	12.05% (9.14%, 14.96%)	13.11% (9.70%, 16.52%)	13.75% (10.49%, 17.01%)	14.12% (11.17%, 17.08%)
Intervention - Control	0.84% (−3.08%, 4.77%)	0.67% (−3.65%, 4.99%)	−0.66% (−4.88%, 3.57%)	−0.80% (−4.55%, 2.94%)

Numbers in parentheses represent 95% confidence intervals estimated from 9,999 bootstrap replicates.

### Objective 2: Effect of STH infections on absenteeism

**Figure 2:**
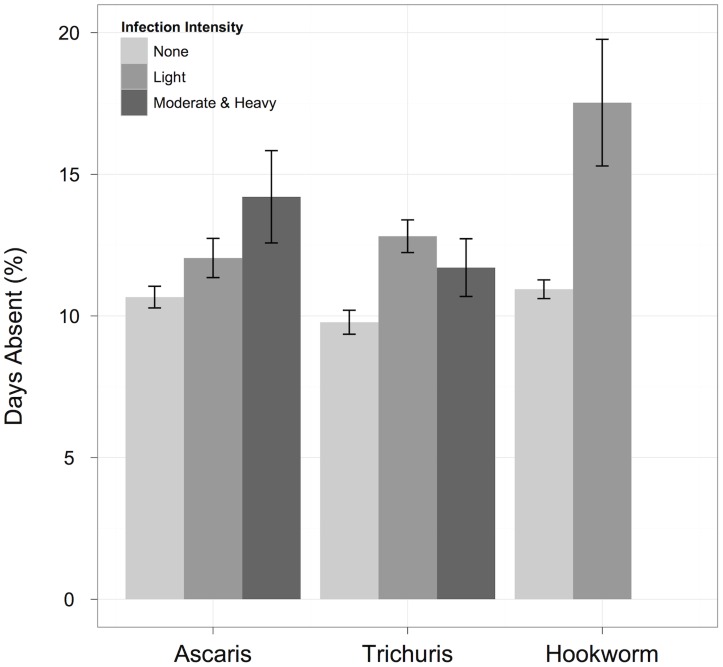
Effect of STH infections on the percentage of schooldays lost. STH infection status was measured four months after deworming, and absenteeism was measured from the day following deworming to the day of follow-up stool specimen collection. Mean (± standard error) percentage of schooldays lost presented for each STH infection category.

STH infections were associated with a significant increase in the percentage of schooldays absent during the four-month follow-up period (Figure 2). After adjusting for SES quartiles, age, and clustering by schools, students who had moderate and heavy *Ascaris* infections at follow-up had missed 2.4% (95% CI: 0.1%, 4.7%) more schooldays than students with no *Ascaris* infections. Similarly, students with light hookworm infections at follow-up had missed 4.6% (95% CI: 1.9%, 7.4%) more schooldays than students with no hookworm infections. *Trichuris* infections at follow-up were also associated with an increase in absenteeism, but the effect was not statistically significant at α = 0.05 (Table 3). Having any STH infection, or having multiple STH infections, were both associated with an increase in absenteeism, but the effect was not statistically significant (Table 4).

**Table 3:** Effect of STH infection intensity, SES and age on the percentage of schooldays absent during a four-month follow-up period, as estimated by three different hierarchical linear regression models, for each STH infection.

Variable	Category	Number of students	Model 1 (*Ascaris*)	Model 2 (*Trichuris*)	Model 3 (hookworm)
*Ascaris*	None	715	Reference		
	Light	282	0.50 (−0.97, 1.96)		
	Moderate-and-Heavy	91	2.38 (0.06, 4.69)*		
*Trichuris*	None	495		Reference	
	Light	481		1.17 (−0.22, 2.56)	
	Moderate-and-Heavy	112		0.37 (−1.94, 2.68)	
Hookworms	None	1062			Reference
	Light	62			4.62 (1.88, 7.36)*
SES	Highest quartile	342	Reference	Reference	Reference
	2nd highest quartile	347	0.63 (−0.97, 2.24)	0.64 (−0.96, 2.25)	0.68 (−0.92, 2.27)
	2nd lowest quartile	178	1.87 (−0.13, 3.86)	1.91 (−0.08, 3.90)	2.04 (0.06, 4.02)*
	Lowest quartile	221	1.98 (0.03, 3.93)*	2.04 (0.10, 3.99)*	2.03 (0.10, 3.96)*
Age		1088	1.69 (1.11, 2.27)*	1.64 (1.07, 2.22)*	1.62 (1.05, 2.20)*

Numbers in parentheses represent 95% confidence intervals.

* Statistically significant effect at a confidence level of α = 0.05

**Table 4:** Effect of infection by any STH species and by multiple STH species on the percentage of schooldays absent during a four-month follow-up period, as estimated by two different hierarchical linear regression models.

Variable	Category	Number of students	Model 1 (Any STH infection)	Model 2 (Polyparasitism)
Any STH infection	No	379	Reference	
	Yes	709	1.15 (−0.26, 2.57)	
STH species	Infected with none	379		Reference
	Infected with one	421		0.88 (−0.63, 2.41)
	Infected with two or three	288		1.64 (−0.10, 3.38)
SES	Highest quartile	342	Reference	Reference
	2nd highest quartile	347	0.63 (−0.98, 2.23)	0.60 (−1.01, 2.21)
	2nd lowest quartile	178	1.83 (−0.16, 3.82)	1.78 (−0.22, 3.78)
	Lowest quartile	221	2.00 (0.06, 3.95)*	1.94 (−0.02, 3.89)
Age		1088	1.66 (1.01, 2.23)*	1.65 (1.07, 2.22)*

Numbers in parentheses represent 95% confidence intervals.

* Statistically significant effect at a confidence level of α = 0.05

**Figure 3:**
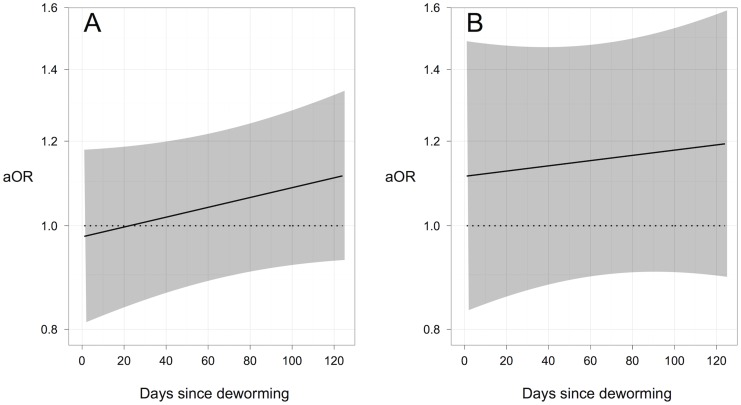
Effect of *Ascaris* infection on the daily odds of absenteeism, following deworming. The daily odds of absenteeism in students with A) light and B) moderate-to-heavy *Ascaris* infections four months after deworming, compared to the odds in students with no *Ascaris* infection four months after deworming, as estimated by a hierarchical logistic regression model. Black lines represent the estimated adjusted odds ratio (aOR), and shaded areas represent 95% confidence intervals.

**Figure 4:**
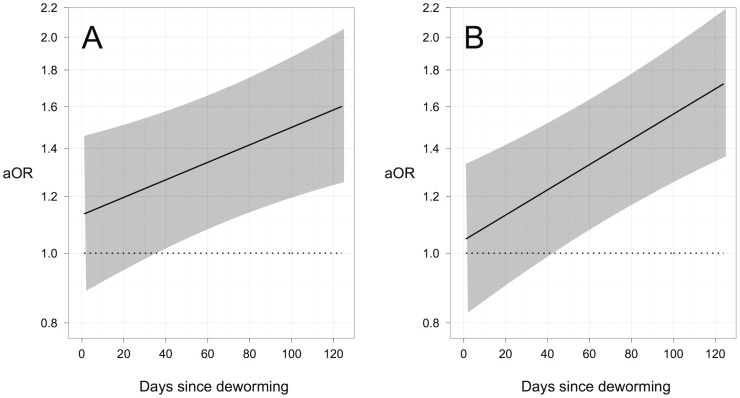
Effect of socioeconomic status (SES) on the daily odds of absenteeism, following deworming. The daily odds of absenteeism in students of A) the second lowest and B) the lowest SES quartile, compared to those in students of the highest SES quartile, as estimated by a hierarchical logistic regression model. Black lines represent the estimated adjusted odds ratio (aOR), and shaded areas represent 95% confidence intervals.

The daily odds of absenteeism slightly increased as the number of days since deworming increased in students who had light and moderate-and-heavy *Ascaris* infections at follow-up. A hierarchical logistic regression model estimated that the adjusted Odds Ratio (aOR) comparing the odds of absenteeism between students with light *Ascaris* infections and no *Ascaris* infections at follow-up increased from 0.98 (95% CI: 0.81, 1.78) on the day following deworming to 1.11 (95% CI: 0.93, 1.34) on the 125^th^ day following deworming (approximately four months post-deworming). Similarly, the aOR comparing the odds of absenteeism between students with moderate-to-heavy *Ascaris* infections and no *Ascaris* infections at follow-up increased from 1.11 (95%CI: 0.83, 1.49) on the day following deworming to 1.19 (95% CI:0.90, 1.59) on the 125^th^ day following deworming (Figure 3). The same model found significant interaction effects between SES quartiles and the number of days since deworming on the odds of absenteeism. The aOR comparing the odds of absenteeism between students in the lowest and highest SES quartiles increased from 1.05 (95% CI: 0.82, 1.33) on the day following deworming to 1.73 (95% CI: 1.36, 2.19) on the 125^th^ day following deworming (Figure 4).

## Discussion

### Objective 1: Effect of a health hygiene education intervention on absenteeism

Four months of a health hygiene education intervention was not enough to reduce absenteeism in Grade 5 students. There was virtually no difference in the mean percentage of schooldays missed among students attending intervention and control schools. However, this result is not surprising given the short follow-up period. Four months might be insufficient for Grade 5 students attending intervention schools to 1) learn about STH prevention; 2) translate their newly acquired knowledge into behavioural changes; 3) have lower STH re-infection rates because of their newly developed healthy behaviours; 4) have fewer illness episodes because of their lower STH re-infection rates; and 5) attend school more frequently because of a lower STH burden. The health hygiene education intervention reduced *Ascaris* infection intensity [21], suggesting that points 1–3, as outlined above, were successfully achieved during the follow-up period. Similarly, a recent trial found that health hygiene education significantly reduced STH infections in Chinese schoolchildren [27]. However, only 34 (6.6%) students from the current study's intervention schools and 57 (10.0%) students from the current study's control schools had moderate-and-heavy *Ascaris* infections (i.e. infection intensity ≥5,000 epg) four months after deworming. This infection intensity threshold is usually associated with an increased risk of *Ascaris*-related morbidity [20]. Too few students from intervention and control schools were therefore at risk of experiencing *Ascaris*-related illness episodes during the four-month follow-up period for a significant effect of the health hygiene education intervention to be observed on absenteeism.

### Objective 2: Effect of STH infections on absenteeism

Absenteeism rates during the follow-up period were higher in students who had STH infections than in those who were still uninfected, four months after deworming. *Ascaris* and hookworm infections were more strongly associated with absenteeism than *Trichuris* infections, which is consistent with earlier findings [13], [14], [17].

The effect measures reported in this study might underestimate the true impact of STH infections on absenteeism for a number of reasons. First, the 18 study schools were beneficiaries of a school lunch program. On any given day, students present at the study schools therefore received a nutritious snack of fortified bread. Such programs offer strong incentives for students to attend school [28]. Infected children who might otherwise stay home because of STH-related illness episodes might therefore attend school to benefit from the lunch program. Second, the single oral dose of 400 mg albendazole used to deworm all students at baseline is very effective against *Ascaris* infections. In fact, a parasitological survey of 267 Grade 5 students, conducted two weeks after deworming, found an *Ascaris* egg reduction rate of 98.8% [29]. It is thus almost certain that students with moderate or heavy *Ascaris* infections four months after deworming did not harbour such intense infections during the entire follow-up period (i.e. they were free of moderate or heavy *Ascaris* infections for part of the four-month follow-up period). The percentage of schooldays absent during follow-up for this group of students might therefore underestimate the typical absenteeism rate of school-age children who are infected over an entire four-month period.

The high likelihood that students with *Ascaris* infections at follow-up were free of infection immediately after deworming also allowed for a novel approach to the analysis of deworming data. After adjusting for appropriate potential confounders, it was found that the daily odds of absenteeism increased more rapidly with time since deworming in students with *Ascaris* infections than in those with no *Ascaris* infections at follow-up (i.e. time since deworming was modifying the effect of *Ascaris* infection intensity on the daily odds of absenteeism). This result, while not statistically significant, nevertheless suggests that *Ascaris* infections are related to absenteeism in school-age children. As Grade 5 students gradually acquired *Ascaris* infections following deworming, they would increasingly be more likely to be absent from school than students who did not acquire *Ascaris* infections during the four-month follow-up period.

### Effect of age on absenteeism

Age was strongly associated with absenteeism. Under normal grade progression in the Peruvian school education system, Grade 5 students should be, on average, 10 or 11 years old. Students older than 11 years are therefore likely to have repeated one or more grades. Grade repetition is strongly associated with poor academic performance, which in turn is strongly associated with high absenteeism rates [5]. In the study population where Grade 5 students' ages ranged from 8 to 16 years, it was therefore not surprising to observe a strong positive relationship between increasing absenteeism and increasing age.

### Effect of socioeconomic status on absenteeism

SES quartile was also strongly associated with absenteeism. Students from the lowest SES quartiles were absent for a greater percentage of schooldays during the four-month follow-up period. Furthermore, the daily odds of absenteeism increased significantly faster in students of the lowest SES quartiles compared to students of the highest SES quartiles, as time since deworming increased. This statistically significant effect modification can be explained by differential patterns of family livelihood. Families of lower SES might call upon their school-age children to work the family's agricultural plot (locally called “chacra”) more frequently than families of high SES. Because deworming occurred near the end of the rainy season, the weather became increasingly favourable to outdoor manual labour as time since deworming increased. It is therefore possible that students of lower SES quartiles spent an increasing number of days at work as the follow-up period progressed.

### Limitations

It must be noted that, while intervention status was randomly allocated, infection status was not. Students who acquired infections during the follow-up period were inherently different from students who were still uninfected, four months after deworming. Although analyses adjusted for potential confounders, it is possible that residual confounding biased the effect measures of STH infections on absenteeism. It must also be noted that this trial was powered to detect a statistically significant effect of the health hygiene education intervention on STH re-infection rates, rather than the relationship between STH infections and absenteeism [21]. Analyses examining outcomes other than re-infection might therefore have been underpowered. Light *Ascaris* infections and both light and heavy *Trichuris* infections had positive but statistically non-significant effects on absenteeism. Effect modification between *Ascaris* infection intensity and time since deworming on the daily odds of absenteeism was also positive but statistically non-significant. With a larger sample size, these effects might have been statistically significant. Other studies [8], [14] have also attempted to measure absenteeism's association with STH infections despite being powered to detect statistically significant differences in health-related outcomes more directly related to STH infections on the hypothetical causal chain. No study has ever been designed and adequately powered for the sole purpose of measuring the effects of STH infections on absenteeism in school-age children. Finally, it must be noted that attendance was not measured directly, and that using the teachers' attendance logs might have introduced measurement bias in the analyses. Assuming that this type of measurement error was random, the reported estimates could have been affected by regression dilution bias and be biased towards the null [30], although it should be noted that such measurement error would be non-differential (i.e. that the same degree of measurement error would be expected to have occurred in both intervention and control schools).

### Conclusion

In conclusion, results of the present study suggest that *Ascaris* infections are related to absenteeism in school-age children. Because the health hygiene education intervention was successful in reducing *Ascaris* infections following deworming [21], it is plausible that this intervention would eventually have lowered absenteeism and also have had a positive impact on future health, had the follow-up period been longer. Incorporating health hygiene education in school-based deworming programs has been found to have positive effects on *Ascaris*-related morbidity [20], but this study found no impact on absenteeism rates. Our results also highlight the importance of allowing a sufficiently long follow-up period before evaluating the health impact of STH-specific health hygiene education interventions in school-age children, especially in areas where STH infection (and subsequent STH-attributable morbidity) is gradually acquired over time.

## Supporting Information

Checklist S1 CONSORT checklist.
(DOCX)

Protocol S1 Study protocol
(DOC)
